# Decontamination of stainless-steel bowls with 80% (w/v) alcohol for 30 s and 60 s: randomized experimental study^*^

**DOI:** 10.1590/1518-8345.4997.3475

**Published:** 2021-09-03

**Authors:** Melissa Santiloni Montanha Ramos, Patricia Leme Paniguel, Terue Sadatsune, Kazuko Uchikawa Graziano, Alessandro Lia Mondelli, Silvia Cristina Mangini Bocchi

**Affiliations:** 1 Secretaria Estadual de Saúde do Estado de São Paulo, Hospital das Clínicas, Botucatu, SP, Brazil.; 2 Universidade Estadual Paulista “Júlio de Mesquita Filho”, Instituto de Biociências de Botucatu, Botucatu, SP, Brazil.; 3 Universidade de São Paulo, Escola de Enfermagem, São Paulo, SP, Brazil.; 4 Universidade Estadual Paulista “Júlio de Mesquita Filho”, Faculdade de Medicina, Botucatu, Botucatu, SP, Brazil.

**Keywords:** Ethanol, Disinfectants, Decontamination, Hospital Equipment and Supplies, Nursing Care, Clinical Nursing Research, Etanol, Desinfetantes, Descontaminação, Equipamentos e Provisões Hospitalares, Cuidados de Enfermagem, Pesquisa em Enfermagem Clínica, Etanol, Desinfectantes, Descontaminación, Equipos y Suministros de Hospitales, Atención de Enfermería, Investigación en Enfermería Clínica

## Abstract

**Objective:**

to compare the efficacy of 80% (w/v) alcohol, rubbed for 30 and 60 seconds, in the manual processing of stainless-steel wash bowls, after cleaning with running water and neutral detergent.

**Method:**

experimental study conducted in a hospital in the state of São Paulo, Brazil, on 50 bowls randomly divided into two groups of 25 bowls each for interventions of 30 and 60 seconds of rubbing with 80% (w/v) alcohol.

**Results:**

based on the microbiological analyses collected, before and after the interventions for both groups, partial efficacy of the disinfectant was observed even when extending rubbing time. In both groups, there was a higher prevalence of survival of *Pseudomonas aeruginosa*, with 14 strains that were resistant to carbapenems, being, specifically, 11 to imipenem and three to meropenem.

**Conclusion:**

stainless-steel bed wash bowls decontaminated for reuse by 80% (w/v) alcohol, after cleaning with running water and neutral detergent, showed to be reservoirs of hospital pathogens. The use of bed wash bowls for patients with intact skin would not have worrying consequences, but considering those with non-intact skin and the contamination of professionals’ hands, the results in this study justify the search for other decontamination methods or the adoption of disposable bed baths.

## Abstract (pt)

**Objetivo:**

comparar a eficácia do álcool 80% (p/v), friccionado por 30 e 60 segundos, no processamento manual de bacias de banho em aço inoxidável, após limpeza com água corrente e detergente neutro.

**Método:**

estudo experimental realizado em hospital do estado de São Paulo, Brasil, com 50 bacias randomicamente distribuídas em dois grupos de 25, para as intervenções de 30 e 60 segundos de fricção com álcool 80% (p/v).

**Resultados:**

das análises microbiológicas coletadas, antes e após as intervenções para os dois grupos, verificou-se eficácia parcial do desinfetante, mesmo ampliando o tempo de fricção. Em ambos os grupos, observou-se maior prevalência de sobrevida de *Pseudomonas aeruginosa*, 14 cepas resistentes a carbapenens, especificamente, 11 ao imipenen e três ao meropenen.

**Conclusão:**

bacias de banho no leito em aço inoxidável, descontaminadas para reuso com álcool 80% (p/v), após limpeza com água corrente e detergente neutro, apresentam-se como reservatórios de patógenos hospitalares. O uso das bacias de banho no leito para pacientes com pele íntegra não teria consequências preocupantes, mas para aqueles com pele não íntegra e pensando na contaminação das mãos dos profissionais, os resultados dessa pesquisa justificam a busca de outros métodos de descontaminação ou a adoção de banho de leito descartável.

## Abstract (es)

**Objetivo:**

comparar la eficacia del alcohol al 80% (p/v), frotado durante 30 y 60 segundos, en el proceso de descontaminación manual de palanganas de baño de acero inoxidable, después de lavarlas con agua corriente y detergente neutro.

**Método:**

estudio experimental realizado en un hospital del estado de São Paulo, Brasil, con 50 palanganas divididas aleatoriamente en dos grupos de 25, para intervenciones de 30 y 60 segundos de frotamiento con alcohol al 80% (p/v).

**Resultados:**

los análisis microbiológicos recolectados, antes y después de las intervenciones para ambos grupos, demostraron efectividad parcial del desinfectante, incluso cuando se extendió el tiempo de fricción. En ambos grupos, se observó una mayor prevalencia de supervivencia de *Pseudomonas aeruginosa*, 14 cepas resistentes a carbapenemas, específicamente 11 a imipenem y tres a meropenem.

**Conclusión:**

las palanganas de baño de cama de acero inoxidable, descontaminadas para su reutilización con alcohol al 80% (p/v), después del lavado con agua corriente y detergente neutro, actúan como reservorios de patógenos hospitalarios. El uso de las palanganas de baño de cama no tendría consecuencias preocupantes para pacientes con la piel íntegra, pero para aquellos cuya piel no conserva su integridad y pensando en la contaminación de las manos de los profesionales, los resultados de esta investigación justifican la búsqueda de otros métodos de descontaminación o la adopción del baño de cama desechable.

## Introduction

Stainless-steel bowls are processable health products (HPs) used in health care services for, among other purposes, the hygiene of bedridden patients. Although automated cleaning and disinfection of these items, using flushing thermal washer-disinfectors is not only more practical, but safer from the point of view of cross- and occupational contamination^(1-2)^, manual decontamination by cleaning with running water and neutral detergent, followed by rubbing with 70% (w/v) alcohol disinfectant, for 30 seconds (30 s), is still a frequent method in our country^(3)^.

Such manual decontamination procedure is based on the bactericidal efficacy of alcohol in various concentrations, and it is concluded that the 30-second exposure time at a concentration of 70% (w/v) is sufficient to eliminate microorganisms^(4)^. Furthermore, wash bowls are considered non-critical items, according to the contamination potential classification^(5)^, which, *a priori*, considers them to be HPs that come into indirect contact with patients’ skin, thus justifying that the usual practice of cleaning and disinfecting them manually with 70% (w/v) alcohol for 30 s is an acceptable procedure as an alternative to automated cleaning and thermal disinfection methods^(6)^.

However, in hospitals, these bowls are commonly used to assist patients of high care complexity, with unhealthy skin and/or colonized intact mucous membranes, which would theoretically have an indication of more stringent procedures than those recommended for non-critical HPs, that is, cleaning followed by lowlevel disinfection^(6)^, justified by their reclassification as semi-critical HPs.

Among the chemical disinfectants currently available, ethyl or isopropyl alcohol is widely used in Brazil and in the world, due to its favorable characteristics, such as low cost and quick and easy access, being, therefore, recommended for procedures for disinfecting inanimate surfaces. One of the pioneering publications recommends the use of alcohol in concentrations of 70 to 90% (w/v) in an exposure time ≥ 60 s^(4)^. These concentration and time parameters - critical points for disinfection - are not consensual in publications, and the minimum concentration of 60% (w/v)^(7)^ and contact time from 30 s to 90 s are also indicated^(7-8)^.

A systematic review on the disinfection of semicritical products using 70% (w/v) alcohol, or in approximate concentrations, points out that such a disinfectant cannot be recommended unrestrictedly for all HPs. However, according to the type of semi-critical material, disinfection can be achieved with and without prior cleaning^(8)^. Although this review did not include an assessment for HPs classified as non-critical, it deductively applies to bowls used in the hygiene care of bedridden patients, as it is less critical.

This investigation is justified considering that, to this date, there is not a single and definitive answer about the safety of alcohol use in the manual decontamination of bowls used in bedridden patients’ body hygiene. Additionally, there is nurses’ technical responsibility to control patients’ cross-contamination by HPs, especially the dissemination of drug-resistant or multidrug-resistant microorganisms. It is noteworthy that bowls are often used for the hygiene of bedridden patients with broken skin as well as for elderly patients who are highly dependent on nursing care to meet their basic human needs, many of whom have undergone or are undergoing invasive procedures (surgeries, catheters) and/or have wounds and infectious processes.

That said, we ask: How efficacious is manual decontamination in the reuse of stainless-steel bowls for bathing bedridden patients by rubbing 80% (w/v) alcohol for 30 s, having previously cleaned them with running water and neutral detergent? Is there a difference in the efficacy of decontamination in increased contact time of 60 s?

As a hypothesis, it was assumed that doubling the 80% (w/v) alcohol contact time would increase the efficacy of the decontamination procedure on these bowls.

In order to answer our questions and test the hypothesis, the following objectives were outlined:

- General: to compare the efficacy of manual decontamination in the reuse of stainless-steel wash bowls by rubbing them with 80% (w/v) alcohol for 30 s and 60 s, after cleaning with running water and neutral detergent;
- Specific: if results indicate the survival of microorganisms, to identify the hospital bacteria isolated after the bowl decontamination procedure, as well as their susceptibility to antimicrobials, compared to previous contamination before decontamination.

## Method

### Design

Randomized experimental single-blinded study, with a before-after design^(9)^, conducted in a single center, according to the Standards for QUality Improvement Reporting Excellence - SQUIRE 2.0^(10)^.

### Site and sample

The study was conducted in a large public hospital with 417 operational beds in the state of São Paulo, from 01/02 to 05/31/2018, on stainless-steel wash bowls used in an inpatient internal-medicine clinic with 19 beds, providing a mean of five baths/day and 150 baths/month, and an estimated mean reuse *per* bowl of 30 times a month.

**Figure 1:**
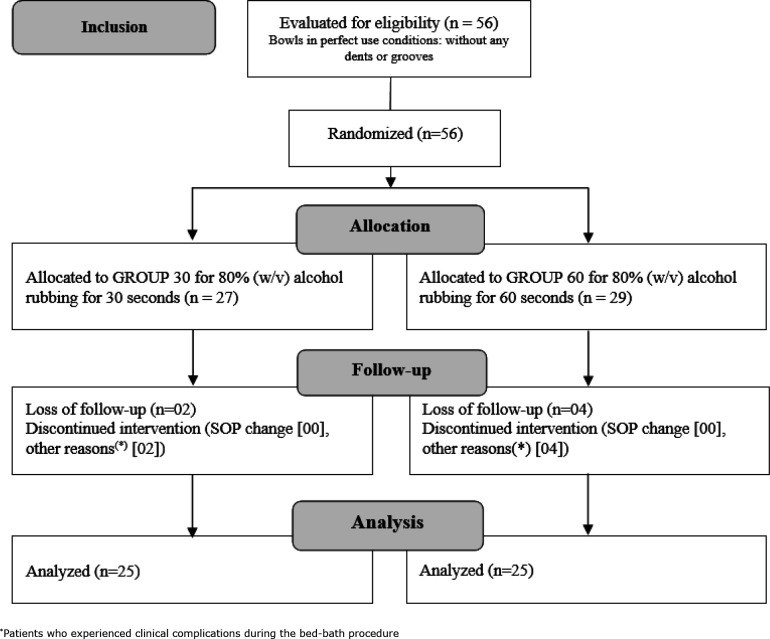
Process of inclusion, allocation, follow-up and analysis of the sample related to a randomized experimental study on the efficacy of decontamination in the reuse of stainless-steel wash bowls by rubbing with 80% (w/v) alcohol for s and 60 s, after cleaning with running water and neutral detergent. Hospital in the state of São Paulo, Brazil, 2018

From these data, a sample with 80% power and 95% reliability was designed, consisting of 50 bowls randomly distributed equally in two groups, as shown in Figure 1, using a paired-proportion test (two moments) and microbiological tests before and after the proposed interventions for each group.

### Variables

- *Characterization of wash bowl users,* by identification [registration number; sex (female; male); age (18 to 59; ≥ 60 years); hospitalization period] and clinical status on the day of data collection [medical diagnosis for hospitalization; number and types of catheters; mechanical ventilation (yes; no); with wounds (yes; no); with infection (yes; no); positive culture (yes; no); isolated microorganism; antibiogram (yes; no); multi-resistant bacteria (yes; no); use of antibiotic therapy (yes; no); type of precaution (standard; contact; droplets; aerosols)];
- *Independent* (antecedent/causal factor): decontamination protocols in the reuse of stainlesssteel wash bowls with by rubbing 80% (w/v) alcohol for 30 s and 60 s, after cleaning with running water and neutral detergent;
- *Dependent* (consequent, outcomes): presence of vegetative hospital bacteria, sensitive or not to antimicrobials, from stainless steel wash bowls, cleaned with running water and neutral detergent, followed by rubbing 80% (w/v) alcohol disinfectant in two steps: 30 s and 60 s;

In order to control the confounding variable related to the concentration of the chemical disinfectant, alcohol sterility and concentration were controlled. For this purpose, two sealed boxes from the same batch of bottles containing 100 ml of alcohol were separated for restricted use in this study. They were labeled as 77º, 70º GL INPM (acronym in Portuguese for *Instituto Nacional de Pesos e Medidas* – National Institute of Weights and Measures) ethyl alcohol. Of these, one bottle was randomly chosen from each box, so that samples were collected for alcoholometry and microbial analysis performed in a laboratory. Results confirmed that the alcohol batch was free from contamination and at a concentration of 80% (w/v), thus justifying the definition of such alcohol concentration for this study.

### Criteria for inclusion, allocation and sample follow-up and analysis

Six bowls used for bed baths at the hospitalization unit were followed-up. All of them were made of stainless steel and had no visible damages, such as dents or grooves.

The bowls were identified alphanumerically, using the initials of the hospitalization unit and the utensil number, for example: CM-1, CM-2, ..., CM-6. Afterwards, they were cleaned with running water and neutral detergent, which was followed by disinfection and storage, according to the procedure used at the institution, that is, the bowls were entirely rubbed with 80% (w/v) for 30 s, according to the protocol for inclusion, allocation and sample follow-up and analysis (Figure 1).

Before beginning data collection, a pilot test was carried out on two bowls, one from each follow-up group, which showed no need for readjustment of the procedural steps of the protocol, including those related to laboratory analyses. Thus, those bowls were included in the sample and designated in the results as “sample 1” (Figure 3) and “sample 2” (Figure 4).

One of the researchers conducted all the datacollection phases, from the randomization process to sample collection before and after cleaning, followed by disinfection, counting on an assistant for support during collections and always in the presence of an observant referee, who followed and strictly verified compliance with all the steps provided for by the protocol in hand, using a form.

The random selection of bowls, that of the patients who would be bathed as well as that of the allocation groups for the 30-s and 60-s procedures were carried out daily, using card draw techniques, in which the cards were duly identified in three brown-paper envelopes, named as follows: the first, “beds”, with the number of beds of bedridden patients with a prescribed bath; the second, “bowls”, with six cards numbered from 1 to 6; the third, related to the “allocation group”, with two cards, one for the time of 30 s and the other for 60 s.

An individual who was unrelated to the study drew the cards from the respective envelopes: “bowl”, “bed” and “allocation group” in the follow-up. If the bowl was not available for reuse, or if the bed was empty, a new card was drawn, and the data collection protocol was followed, as described:

1. researcher - distribute the drawn bowls to the nursing technicians responsible for providing the bed bath to the respective patient, instructing them, to hand the bowls over to the researcher after the procedure is finished, still containing the bath water, to dispose of it in the utility room and, subsequently, collect the first sample for microbiological culture;
2. researcher - proceed to hand washing and put on sterile gloves to receive the bowl;
3. researcher - use an aseptic technique to collect a microbiological sample by scanning the whole internal area of the bowl, using two sterile, overlapping compressed hydrophilic-gauze layers, and sliding them clockwise and with uniform movement, covering the whole internal circumference of the bowl, the flap, the sides and, finally, the bottom;
4. researcher - deposit the gauze in a 100-mL Schottglass vial, with 50 mL of sterile Brain Heart Infusion (BHI) culture medium;
5. assistant - close the Schott-glass vial hermetically, identifying it with the following information: sample number (1, 2, 3, 4, 5, ..., 50), allocation group (Code A: 30 s or Code B: 60 s), bowl number (1, 2, 3, 4, 5, 6), follow-up phase (before), date and time of collection;
6. researcher - discard gloves and wash hands to put on Personal Protective Equipment (PPE);
7. researcher - moisten the bowl and sponge with running water, pouring neutral detergent into the sponge. Then, wash the bowl, rubbing it with the sponge over its whole internal and external surfaces, then rinse it with running water until all the apparent detergent is removed;
8. researcher - position the bowl on a bench with cleaned wit 80% (w/v) alcohol, lined with a sterile double field in order to drain excess water;
9. researcher - take off rubber gloves and clean hands;
10. researcher/assistant - researcher: put on sterile gloves to dry the bowl using sterile surgical compressed gauze provided by an assistant, and then support the bowl on a bench lined with a sterile double field;
11. researcher - take off gloves, wash hands and puton sterile gloves;
12. auxiliary/researcher - assistant: open a package of sterile surgical 25-cm x 28-cm compressed gauze for the researcher to take it and then soak it with 50 mL of 80% (w/v) alcohol from a batch that has been previously evaluated by alcoholometry;
13. researcher/assistant - researcher: slide the compressed 80% (w/v) alcohol-soaked gauze, rubbing it along the whole bowl in a clockwise, continuous and uniform motion, beginning from the flaps, then proceeding to internal sides and finishing at the bottom, as well as over the whole external area of the bowls allocated in one group for 30 s and of those in the other group for 60 s, controlled by an assistant using a seconds timer;
14. researcher - place the bowl on a sterile field spread over a dry bench, after cleaning it with 80% (w/v) alcohol);
15. researcher - take off and discard gloves and wash hands in order to put on sterile gloves;
16. researcher/assistant - researcher: ask the assistant to open a sterile hydrophilic gauze wrapper and take two overlaps, so that he/she can moisten them with 10 mL of sterile 0.9% saline solution (10 mL-ampoule) in order to collect a biological sample from the bowl by sliding the gauze clockwise, beginning from the flap, proceeding to the sides and completing the sweeping on the bottom so as to perform the procedure on the whole internal area of the bowl;
17. researcher/assistant - researcher: deposit gauze in a Schott-glass vial with 50 mL of sterile BHI broth of the OXOID^®^ brand, opened by the assistant, who should close and identify it with the following information: allocation group (Code A: 30 s or Code B: 60 s), bowl number (1, 2, 3, 4, 5, 6), sample number (1, 2, 3, 4, 5, 6,7, 8, …, 50), follow-up phase (after), date and time of collection;
18. researcher - wash hands after taking off and discarding gloves;
19. assistant - accommodate the sample vials in an appropriate container for transportation and send them to the microbiological research laboratory, immediately after collection completion.

Before the vials were incubated at FANEM^®^ brand, at the microbiology laboratory, with periodic temperature control, cultures of the previous samples related to the disinfection procedure were performed, aiming at the numerical estimation of microorganisms (direct test). For such test, the vials were shaken vigorously for 30 s, and 10-µL aliquots were spread in Petri dishes by using an L-shaped glass rod. The dishes contained specific culture media for Gram-negative (McConkey agar) and Gram-positive (Columbia CNA agar and sheep blood) bacteria. Then, the vials and plates from the direct test were incubated in an oven at 35 ± 1 °C for 24 to 48 h. After 24 hours, Colony-Forming Units (CFUs) were counted, and the direct-test plates were analyzed.

Streak plate method was performed on vials showing broth turbidity (positive culture) using different culture media, all of them being of the OXOID^®^ brand: MacConkey Agar (for Gram-negative bacteria), blood agar with Columbia CNA base (for Grampositive bacteria), Cetrimide Agar (for *Pseudomonas aeruginosa*), Mannitol Salt Agar (for *Staphylococcus aureus*), Slanetz-Bartley Agar (for Enterococci) and Sabouraud Dextrose Agar added with chloramphenicol (for yeasts). Those plates were incubated at 35 ± 1°C for 24 to 48 h, in order to isolate and identify hospital microorganisms.

Colonies from different culture media were identified by conventional phenotypic tests^(11)^.

The Antimicrobial Susceptibility Test (AST) by agar disc-diffusion^(12)^ was used to evaluate the profile of bacteria isolated from wash bowls, and the reading was based on the Clinical and Laboratory Standards Institute (CLSI-2017)^(13^). In order to perform the antibiograms, discs of the CEFAR^®^ brand were used, namely SENSIFAR - ANTIBIOGRAMA CLSI/BrCAST, before their expiry dates had passed. For enterobacteria, the following drugs were used: amikacin, cefepime, ceftriaxone, cefuroxime, ciprofloxacin, ertapenem, gentamicin, imipenem, meropenem, piperacillin/tazobactam, ampicillin and cefoxitin. For *Pseudomonas aeruginosa* and *Stenotrophomonas maltophilia*, the drugs were: amikacin, cefepime, ceftazidime, ciprofloxacin, colistin, gentamicin, imipenem, meropenem, piperacillin/ tazobactam. For *Acinetobacter baumannii*, the antimicrobials were the same as those used for the *Pseudomonas* group, with the addition of three drugs: ceftriaxone, tigecycline and ampicillin/sulbactam. For *Enterococcus faecium* and *Enterococcus faecalis*, resistance analysis to vancomycin was performed using plates with bile-esculin agar plus 6 µg/mL of vancomycin.

The efficacy of the intervention of manually processing wash bowls was considered to be the absence of hospital bacteria in the vegetative form and, in the presence of such bacteria, it was found to be ineffective, since the bowl played a role in promoting the spread of microorganisms in both sample groups.

For this study, hospital microorganisms were understood to be those found in epidemiological profile of the investigation site: *Acinetobacter baumannii*; *Candida albicans*; *Candida glabrata*; *Candida tropicalis*; *Citrobacter freundii*; *Citrobacter koseri*; *Enterobacter cloacae*, *Enterobacter agglomerans*, *Enterobacter aerogenes*; *Enterococcus faecalis*; *Enterococcus faecium*; *Escherichia coli*; *Klebsiella pneumoniae*; *Morganella morganii*; *Proteus mirabilis*; *Pseudomonas aeruginosa*; *Serratia marcescens*; *Staphylococcus aureus*; *Stenotrophomonas maltophilia*.

Six bowl samples were excluded from the follow-up due to protocol interruptions resulting from patients’ clinical changes during the bed bath. Such bowls were replaced according to the inclusion criteria, until completing the outlined sample, as shown in Figure 1.

It is noteworthy that there was no double blinding relative to the researchers and operators in charge of the bed-bathing interventions with the participants/ bowls. However, the results from the collected cultures were only known to the researchers and the other participants involved in the study after data collection was completed. The researchers did not participate in the microbiological analyses and the microbiologists who processed the samples were unaware whether the material under analysis belonged to the 30-s or the 60-s group.

### Data analysis

The Stata software, version 14, was used for statistical analysis. The chi-square test was used for variables characterizing bowl users: gender, age group, period of hospitalization, number of catheters, wounds, diagnosis of infection, multidrug-resistant microorganism, contact isolation, undergoing antibiotic therapy and bowl classification according to contamination risk and potential^(5)^. For variable respiration type, Fisher’s Exact Test was used, and in order to evaluate microorganisms reduction after interventions in the 30-s and 60-s Groups as well as to compare the statistical significance of such reduction between them, the generalized linear regression model, namely the Wald test, was applied, with p<0.05 being considered significant.

### Ethical procedures

This project was conducted after approval by the Research Ethics Committee (CAAE: 68181017.8.0000.5411, Report: 2.426.902) and the signature of an Informed Consent Form for participation in the study by bowl users, and upon their incapacity, by a responsible relative.

The study was conducted with financial support from The São Paulo Research Foundation - FAPESP (funder 1) and from *FW Indústria e Comércio de Produtos de Higiene* (funder 2), which did not interfere in the conduct of the research at any time.

## Results

The analyses showed homogeneity in the random allocation of stainless-steel wash bowls in the followup strata, 30-s and 60-s groups, as they did not show statistically significant differences between the variables related to the clinical characteristics of bowl users and, consequently, to their classification, according to the degree of risk for infection after use^(5)^.

**Figure 2:**
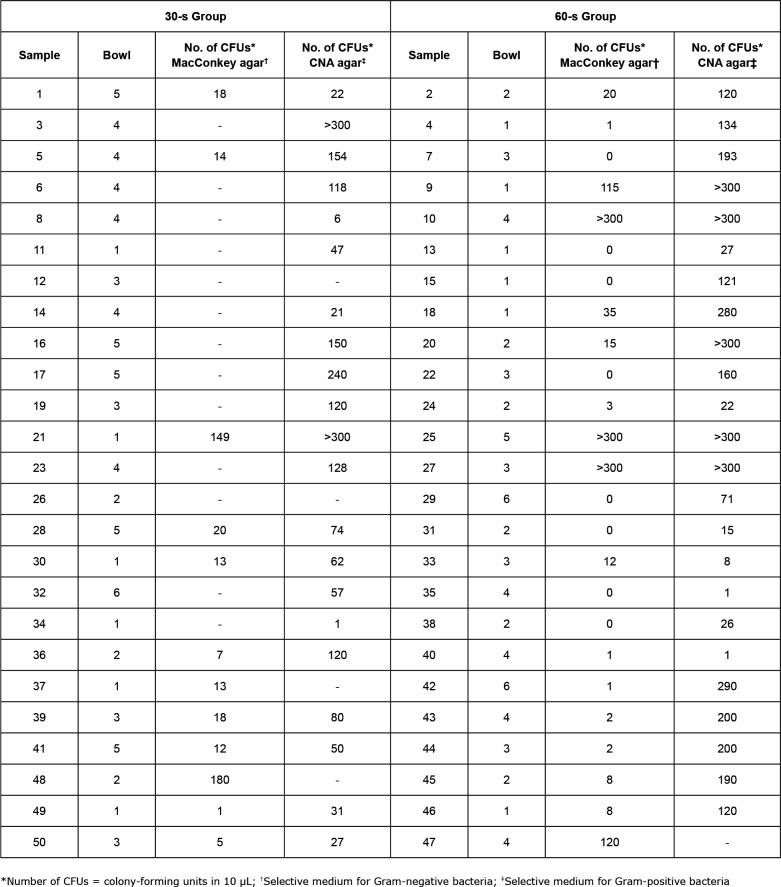
Semi-quantitative pre-incubation analysis of microbiological samples from stainless-steel wash bowls randomly allocated in the 30-s and 60-s Groups, collected before decontamination by 80% (w/v) alcohol, preceded by cleaning using running water and neutral detergent. Hospital in the state of São Paulo State, Brazil, 2018

**Figure 3:**
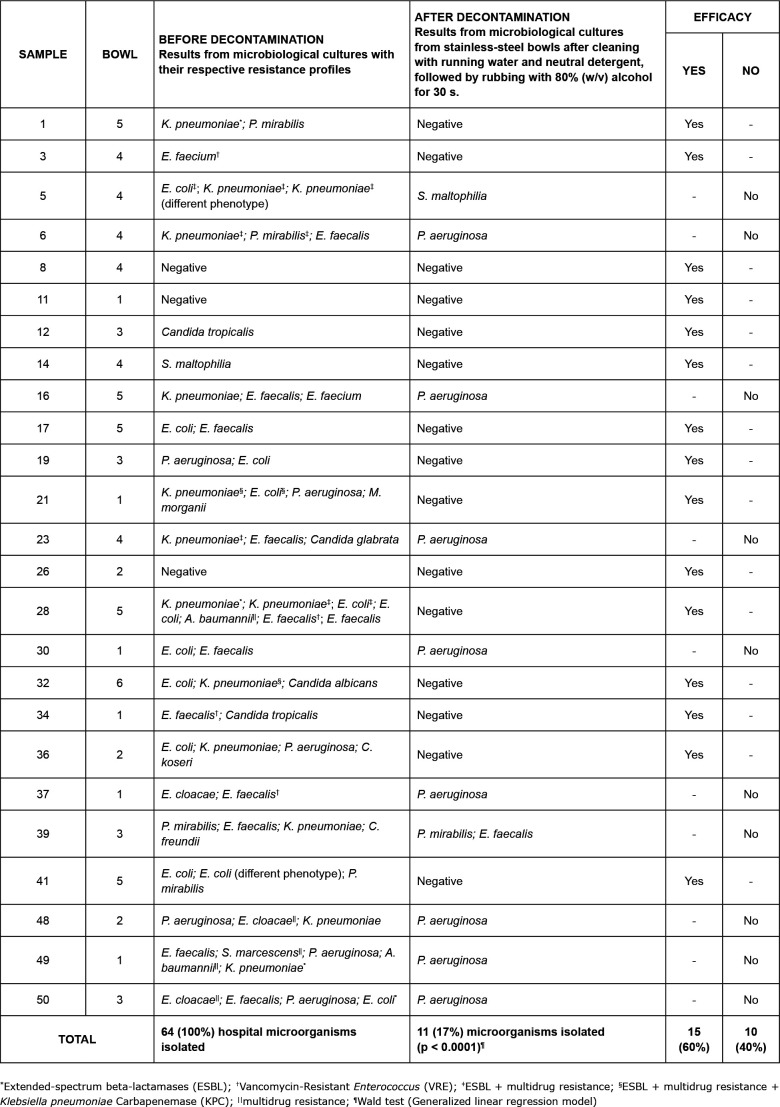
Decontamination efficacy for reuse of stainless-steel wash bowls by comparing the results of microbiological cultures with antimicrobial-resistance profiles, collected before and after rubbing with 80% (w/v) alcohol for 30 s, preceded by cleaning with running water and neutral detergent. Hospital in São Paulo state, Brazil, 2018

**Figure 4:**
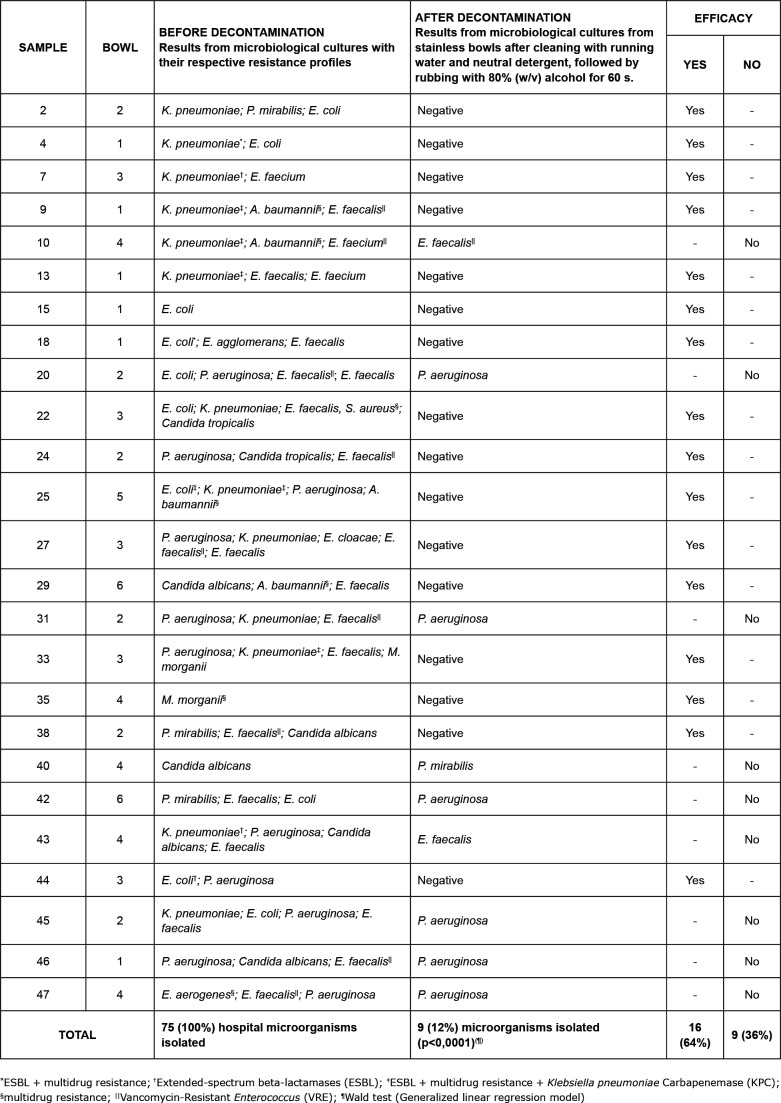
Decontamination efficacy for reuse of stainless-steel wash bowls by comparing the results of microbiological cultures with antimicrobial-resistance profiles, collected before and after rubbing with 80% (w/v) alcohol for 60 s, preceded by cleaning with running water and neutral detergent. Hospital in São Paulo state, Brazil, 2018

Of the total number of analyzed bowls (50, 100%), equally distributed in the 30-s and 60-s Groups, the majority are classified as semi-critical material (100%; 98%; p=0.312), considering the degree of risk for infection that they offered, according to the clinical characteristics of their users at the time of data collection, as well as the microbiological and antimicrobial-resistance profile of hospital strains isolated in cultures of samples collected from such bowls immediately after the bath water was discarded (Figures 2 to 4).

Most users were elderly (88%; 80%; p=0.440) using from one to five catheters (100%; 96%; p=0.312), diagnosed with infection (80%; 80%; p=1.000) and isolated multidrug-resistant microorganisms (40%; 28%; p=0.370), undergoing antibiotic therapy (88%; 84%; p=0.684) and in contact isolation (40%; 32%; p=0.556).

**Figure 5:**
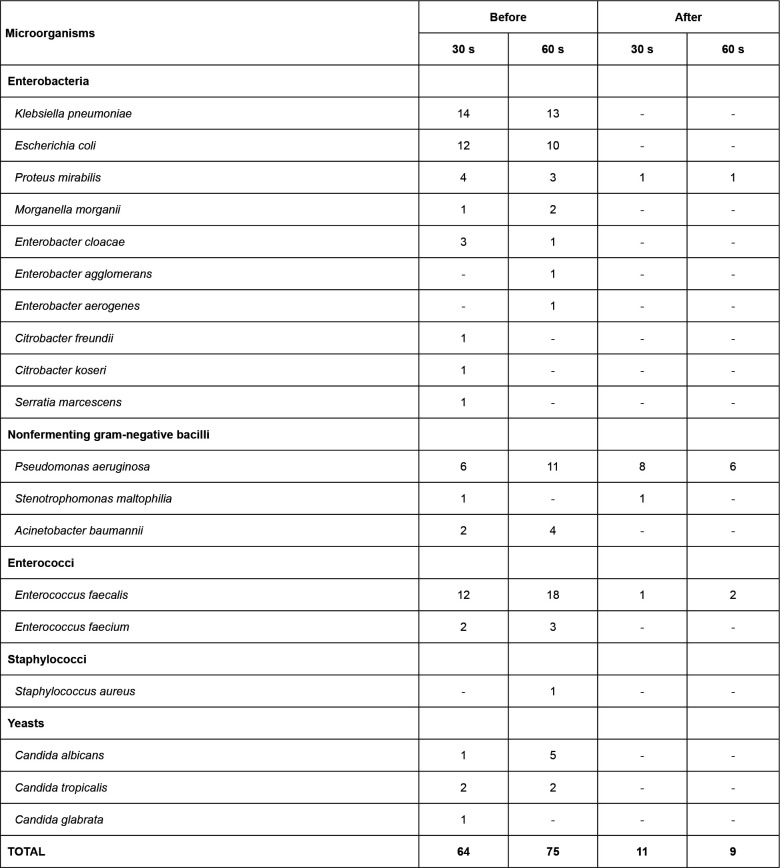
Distribution of hospital microorganisms isolated from stainless bed wash bowls before and after disinfection using 80% (w/v) alcohol for 30 s and 60 s, preceded by cleaning with running water and neutral detergent. Hospital in São Paulo state, Brazil, 2018

The other results are summarized in Figures 2 to 5.

## Discussion

The results from this investigation have refuted the initial hypothesis in the study. It was found that rubbing with 80% (w/v) alcohol, even when doubling the application time from 30 s to 60 s after cleaning, could not decontaminate the stainless-steel bowls used for patients’ bed bath, as it achieved only statistically significant reduction (p=0.0001) in the bacterial load.

The following were recovered as hospital bacteria: *Pseudomonas aeruginosa*, *Proteus mirabilis*, *Enterococcus faecalis* and *Stenotrophomonas maltophilia*, some of which are resistant to antimicrobials.

Considering alcohol an intermediate-level disinfectant with mycobacterial, virucidal, fungicidal and vegetative bactericidal action, bacteria in vegetative form should have been eliminated. This fact raises concern, not only about the patients who are cared for with the use of contaminated HPs, but also about the health professionals who handle them, with the risk of making them reservoirs of such microorganisms, if hand-washing protocols are not complied with.

The expectation that doubling the bowl rubbing time using 80% (w/v) alcohol, from 30 s to 60 s, would impact the efficacy of the disinfectant has not been confirmed, since, when comparing rubbing during the aforementioned times, there was no statistically significant difference between groups (p=0.254).

Some unexpected findings must be discussed, such as the prevalence of *Pseudomonas aeruginosa* recovery, both in the 30-s and in the 60-s groups, which, in some cases, was only inexplicably isolated in a microbiological sample after the disinfection procedure (Figures 3, 4 and 5). As a result, it was conjectured that biofilm may exist on the bowls and that such microorganisms may have developed tolerance to alcohol, as happened with *Enterococcus* ^(14)^. Regarding biofilm, the fact is that these microorganisms have great capacity to form it. A study on 45 bacterium strains isolated from cockroaches captured in hospitals showed the capacity of biofilm formation by all strains, on which the bactericidal effect of alcohol decreased to 60% in the case of adherent bacteria, when compared to 100% effect on free cells^(15)^. Additionally, from the literature review, it was assumed that the residual action of alcohol disinfection itself contributes to increase the formation of biofilm produced by *P. aeruginosa,* more specifically on Psl and Pel synthesis, considered to be exopolysaccharides from such bacterium^(16-17)^. This may explain the fact that the bacterium appeared only in the microbiological analysis performed after the disinfection procedure, in the hypothesis that the bowls analyzed had biofilm.

Another noteworthy aspect in the results is the microbial load shown by the 50 basins. Before decontamination, 47 (94%) were contaminated with microorganisms of hospital importance, comprising 139 strains of hospital microorganisms, with 51 (37%) distributed in five possible groups of resistance to antimicrobials: (A) Multidrug resistant - MR (12; 23%); (B) Extended-spectrum betalactamases - ESBL (7; 14%); (C) ESBL + multidrug resistance (10; 20%); (D) ESBL + multidrug resistance + *Klebsiella pneumoniae* Carbapenemase - KPC (9; 18%); (E) Vancomycin-Resistant *Enterococcus* - VRE (13; 25%) (Figures 3 and 4). This finding reinforces the importance of using standard precautionary principles by those who will perform decontamination. It is known that, in some service routines, this responsibility is delegated to workers without health care training, such as those who work in the hospital cleaning service.

The efficacy of a chemical disinfectant is multifactorial, involving determining factors for microbicidal action, such as: number, location and innate resistance of microorganisms, time and temperature of exposure, concentration and potency, as well as chemical and physical factors, organic and inorganic matter and biofim^(7)^ and, certainly, these factors justify the divergent results in studies and in the clinic practice involving alcohol.

The results in this study show scientific evidence that stainless-steel wash bowls are playing a role as fomites in the spread of strains of hospital microorganism strains that are resistant to antimicrobials, when processed with 80% (w/v) ethyl alcohol, even when it is rubbed according to recommended concentrations and periods of time^(4)^.

Ethyl or isopropyl alcohol has been indicated for intermediate and low-level disinfections, on smooth and hard surfaces, with a minimum exposure time of 60 s^(4,7)^, in concentrations between 70 and 90%^(4)^, the minimum concentration found in the literature being 60% (w/v)^(7)^.

In Brazil, bed-bath bowls are stainless and reused, and they usually undergo a decontamination procedure of 30-s rubbing with 70% (w/v) ethyl alcohol, after previous cleaning with running water and neutral detergent, followed by drying^(3)^. Such time of exposure to ethyl alcohol is based on experimental research on suspended microorganisms, published in the 1980s^(4)^ and ratified by several studies. The universal recommendation to clean one’s hands with 70% (w/v) alcohol also testifies to the belief in the efficient microbicidal action of alcohol in this concentration^(18-19)^.

Considering: (a) the partial efficacy of 80% (w/v) alcohol and the non-significant difference between 30 s and 60 s of rubbing with that disinfectant for decontamination in the reuse of stainless-steel wash bowls; (b) the varied clinical characteristics of their users and the microbiological and antimicrobial-resistance profiles of the organisms present in the bowls after discarding the water; (c) the potential that such bowls have to play the role of fomites in the dissemination of important hospital strains for epidemiological surveillance, since they are still classified as non-critical material and, as shown by complete genomic sequencing and exemplified by *K. pneumoniae* transmission, that such transmission is cross-linked and non-environmental^(20)^; (d) the need to reclassify stainless-steel wash bowls as semicritical material when in use for patients with unhealthy skin and, therefore, requiring high-level disinfection, which can be achieved by automated means, such as thermal disinfectors^(21)^ that guarantee process uniformity and prevent contact of chemical products with those who process the materials; (e) the scarcity of research on the efficacy of decontamination of wash bowls by thermal disinfectors; (f) the scientific evidence on the 90% microbiological efficacy of disposable bed baths, as compared to 20% of conventional baths, among other benefits^(3)^, there is a need to reclassify stainless-steel wash bowls as semi-critical HPs, when in use for patients with unhealthy skin or with invasive devices, such as catheters and probes and, therefore, requiring high-level disinfection. A relatively simple and practical measure is the replacement of the conventional bed-bathing technique for disposable methods^(3)^, in this case weighting issues regarding costs and environmental sustainability.

As a limitation to this study, the fact that a control group was not created, so as to compare with disinfection efficacy by thermal disinfectors, was considered.

Finally, the authors understand that one of the contributions from this study is the fact that alcohol efficacy as a disinfectant cannot be considered in an uncritical way.

## Conclusion

There was not total elimination of vegetative bacteria from the bed-bath wash bowls decontaminated by cleaning with running water and neutral detergent, followed by rubbing with 80% (w/v) alcohol for 30 s and 60 s, with predominant recovery of *Pseudomonas aeruginosa*, including those resistant to antimicrobials, which refuted the initial hypothesis in the study.

Although alcohol is an intermediate-level chemical disinfectant, which, is theoretically a mycobactericide, virucide, fungicide and vegetative bactericide, the results in this investigation have not confirmed this spectrum of microbial action, leading to the risk for disinfected HPs to be characterized as fomites in the context of crosscontamination.

The use of bed-bath wash bowls for patients with intact skin would not have worrying consequences, but it would for those with non-intact skin, thus requiring other decontamination methods or the adoption of disposable bed baths. Additionally, the handling of contaminated bowls contributes to the spread of microorganisms when there is a failure to adhere to hand-hygiene recommendations.
